# Treatment of allergic rhinoconjunctivitis: a review of the role of topical levocabastine

**DOI:** 10.1155/S0962935195000822

**Published:** 1995-05

**Authors:** R. Gerth van Wijk

**Affiliations:** 1 Academisch Ziekenhuis Dijkzigt Rotterdam The Netherlands

## Abstract

Lcocabastine is an extremely potent and highly selective H_1_-receptor antagonist which has been specifically developed as eye drops and nasal spray for the treatment of allergic rhinoconjunctivitis. Clinical experience to date suggests that this topical antihistamine is at least as effective as other current first-line therapeutic approaches for the treatment of this condition, including oral H_1_-receptor antagonists and sodium cromoglycate. Onset of action is rapid, with clinical effects apparent within minutes of instillation. Moreover, duration of action is sufficiently long to permit a convenient twice-daily dosing regimen. Topical levocabastine is well tolerated with an adverse-effect profile comparable with that of placebo and sodium cromoglycate. As might be expected from the route of drug administration, application site reactions are the most frequent adverse effect associated with levocabastine eye drops and nasal spray with an incidence comparable with that seen in placebotreated controls. The availability of effective and well-tolerated topical antihistamines, such as levocabastine, is an important advance which broadens the range of therapeutic approaches available for the clinical management of allergic rhinoconjunctivitis. Levocabastine appears to be an attractive alternative to oral antihistamines as a first-line therapeutic option for the treatment of this atopic condition.


Mediators of Inflammation 4, S31–S38 (1995)

### Treatment of allergic rhinoconjunctivitis: a review of the role of topical levocabastine

R. Gerth van Wijk

Academisch Ziekenhuis Dijkzigt, Rotterdam, The Netherlands

**LEVOCABASTINE is an extremely potent and highly selective $H_1$-receptor antagonist which has been specifically developed as eye drops and nasal spray for the treatment of allergic rhinoconjunctivitis. Clinical experience to date suggests that this topical antihistamine is at least as effective as other current first-line therapeutic approaches for the treatment of this condition, including oral $H_1$-receptor antagonists and sodium cromoglycate. Onset of action is rapid, with clinical effects apparent within minutes of instillation. Moreover, duration of action is sufficiently long to permit a convenient twice-daily dosing regimen. Topical levocabastine is well tolerated with an adverse-effect profile comparable with that of placebo and sodium cromoglycate. As might be expected from the route of drug administration, application site reactions are the most frequent adverse effect associated with levocabastine eye drops and nasal spray with an incidence comparable with that seen in placebo-treated controls. The availability of effective and well-tolerated topical antihistamines, such as levocabastine, is an important advance which broadens the range of therapeutic approaches available for the clinical management of allergic rhinoconjunctivitis. Levocabastine appears to be an attractive alternative to oral antihistamines as a first-line therapeutic option for the treatment of this atopic condition.**

**Key words:** Allergic rhinoconjunctivitis, $H_1$-receptor antagonist, Histamine, Levocabastine, Topical antihistamine

### Epidemiology and Aim of Therapy

Allergic rhinoconjunctivitis is a common atopic condition which is frequently encountered in clinical practice, with current estimates suggesting that as many as 22% of the general population may be affected.[1] Available epidemiological data suggest that the incidence of this atopic disorder is increasing,[1,2] particularly in urban areas, possibly as a result of environmental pollution.[2–4] The relationship between air pollution and the prevalence of allergic disease is, however, complex. Analysis of the prevalence of respiratory diseases and atopic disorders in German children has revealed that the prevalence of allergic disorders was lower in the former East Germany than in West Germany in spite of higher pollution levels.[5] Characteristic clinical manifestations include nasal itching, sneezing, rhinorrhoea and congestion, often accompanied by ocular symptoms of lacrimation, redness and itching. Causative allergens are diverse and include grass, tree and weed pollens, fungal spores, house dust mite and animal dander.

The medical and socioeconomic impact of allergic rhinoconjunctivitis is often underestimated. Although rarely associated with long-term clinical complications, symptoms may be sufficiently severe to impact on the patient's quality of life, with almost all patients experiencing a degree of sleep impairment, limitation of normal daily activities and emotional distress.[6] These findings are supported by data from the US Department of Health which reveal that allergic rhinoconjunctivitis accounts for more than 2 million lost school days and 3.5 million lost work days every year in the USA alone.[7]

Treatment of allergic rhinoconjunctivitis should not only be aimed at direct amelioration of symptoms. The subsequent inflammation after allergen exposure may induce non-specific hyperreactivity and nasal priming.[8,9] Reduction of this inflammation may therefore be expected to interrupt the vicious circle of early and late sequelae of allergen exposure, including nasal hyperreactivity. Indeed, it has been demonstrated that treatment of the nose may have a beneficial effect on lung function and bronchial hyper-

© 1995 Rapid Communications of Oxford Ltd responsiveness in patients with concurrent asthma.[10,11]

The fundamental approach to the treatment of allergic rhinoconjunctivitis is environmental control, combined with appropriate antiallergic drug therapy and, in selected cases, specific immunotherapy. Levocabastine is a novel $H_1$-receptor antagonist which has been specifically developed for the topical treatment of allergic rhinoconjunctivitis. The aim of this paper is to review the clinical experience of this topical antihistamine available to date, with particular reference to the implications for patient management.

### Pathophysiology: the Role of Histamine

Our understanding of the pathophysiology of allergic rhinoconjunctivitis has increased considerably in recent years revealing a number of potential targets for pharmacological intervention. Therapeutic approaches available for the clinical management of this atopic condition include $H_1$-receptor antagonists, vasoconstrictors, corticosteroids, and mast cell stabilizers, such as sodium cromoglycate. Although multiple inflammatory mediators have been implicated in the pathogenesis of allergic rhinoconjunctivitis, histamine appears to play a prominent role.[12] Experimental allergen challenge studies have revealed that histamine is the only mediator which produces the full spectrum of clinical manifestations of the acute allergic reaction when applied to the nasal and ocular mucosa. The available pathophysiological evidence therefore supports the current clinical practice for use of $H_1$-receptor antagonists as a primary treatment option.[13]

The efficacy and tolerability of oral antihistamines in the treatment of allergic rhinoconjunctivitis is well documented..14 However, although the reported incidence of adverse reactions such as sedation is minimal with newer drugs of this class, the potential for unwanted systemic effects, as exemplified by the arrhythmic effects seen with certain oral antihistamines, clearly exists.[15] In addition, as might be expected from the route of drug administration, onset of action with oral antihistamines is relatively slow. Peak antihistaminic activity is typically not observed for several hours,[14] necessitating administration prior to allergen exposure for maximum clinical benefit.

### Rationale for Topical Therapy

Treatment for allergic rhinoconjunctivitis need not necessarily be systemic. Topical therapy is possible due to the accessibility of the affected tissues. A topical agent may be expected to have a number of advantages over an orally administered drug, including a faster onset of action, since it is applied directly to the affected site, and a reduced potential for systemic adverse effects. Until recently, however, topical administration of $H_1$-receptor antagonists has not been feasible as the available agents have not been sufficiently potent to permit single agent therapy. Topical treatment for allergic rhinoconjunctivitis was therefore limited to sodium cromoglycate, vasoconstrictors and corticosteroids.

The mast cell stabilizer, sodium cromoglycate, is both effective and well tolerated for the prophylaxis of allergic rhinoconjunctivitis. However, it has a slow onset of action and may take several days to achieve full therapeutic effects.[16] As a result, treatment should preferably be initiated prior to allergen exposure and maintenance therapy is essential in patients with frequent symptoms. As sodium cromoglycate requires frequent instillation, sometimes as often as six times daily, patient compliance with a long-term maintenance regimen is likely to be problematic.

Like sodium cromoglycate, topical vasoconstrictor and antihistamine/vasoconstrictor combinations are also limited by the need for frequent instillation. Furthermore, although these topical preparations provide rapid symptomatic relief, they are only suitable for short-term use. Long-term administration is associated with rebound vasodilatation which may result in rhinitis and conjunctivitis medicamentosa.[17,18] These agents should, therefore, generally not be used for more than 5 to 7 days consecutively.

Topical corticosteroids are highly effective anti-inflammatory agents, however they also have a slow onset of action, typically taking several days to achieve full therapeutic effects.[13] Consequently, these agents are most effective when administered prophylactically and treatment should preferably be initiated prior to the onset of symptoms for maximum clinical benefit. Furthermore, while intranasal corticosteroids are generally well tolerated and the risk of suppression of the hypothalamic–pituitary–adrenal axis is low following topical application of these drugs, long-term ocular administration should generally be avoided due to the potential for serious adverse effects including glaucoma, cataracts and severe corneal infections.[19]

Two topical antihistamine preparations are now available for the treatment of allergic rhinoconjunctivitis, levocabastine and azelastine. Levocabastine is an extremely potent and highly selective $H_1$-receptor antagonist which has been specifically developed as eye drops and nasal spray for the topical treatment of allergic rhinoconjunctivitis.[20,21] Azelastine appears to possess other anti-allergic properties in addition to its antihistaminic activity which may be of benefit to patients with this atopic condition, but is currently only available as a nasal spray necessitating combination therapy with other anti-allergic agents in patients with concurrent ocular symptoms.

### The Efficacy of Levocabastine in Adults

Levocabastine is the most potent antihistamine available to date, expressing antihistaminic activity at doses lower than 0.002 mg/kg, with *in vitro* data derived from the compound 48/80 lethality test in rats suggesting that it is 15 000 times more potent than chlorpheniramine and 1000 times more potent than azelastine.[22,23] Levocabastine has a highly specific binding affinity for $H_1$-receptors, with no evidence of anticholinergic activity at doses considerably in excess of therapeutic concentrations. Detailed pharmacokinetic analysis demonstrates that levocabastine is well suited to the topical treatment of allergic rhinoconjunctivitis, with the clinical benefits seen with this agent being predominantly mediated by local antihistaminic effects in the ocular and nasal mucosa.[24,25]

Histamine and allergen challenge studies have shown that levocabastine is a potent inhibitor of the allergic response in the human eye and nose.[26–31] Onset of action is rapid, with significant symptomatic relief typically seen within minutes of administration.[28,30] Duration of action is sufficiently long to permit a convenient twice daily dosing schedule.[32]

A comprehensive programme of clinical trials has been undertaken to assess the therapeutic efficacy of topical levocabastine for the treatment of allergic rhinoconjunctivitis in adults. Key findings from studies published to date are summarized below.

*Levocabastine and oral antihistamines:* A number of clinical trials have assessed the comparative efficacy of levocabastine and second-generation oral antihistamines. Levocabastine eye drops (0.5 mg/ml: one drop in each eye twice daily) and nasal spray (0.5 mg/ml: two puffs in each nostril twice daily) have been shown to be at least as effective as oral terfenadine (60 mg twice daily) for the treatment of seasonal allergic rhinoconjunctivitis in double-blind studies involving more than 350 patients to date.[33] A number of statistically significant differences in favour of the topical antihistamine have also been reported in some trials.[34,35] Patient evaluations after 8 weeks of treatment have shown that the effect of therapy on ocular symptoms was excellent or

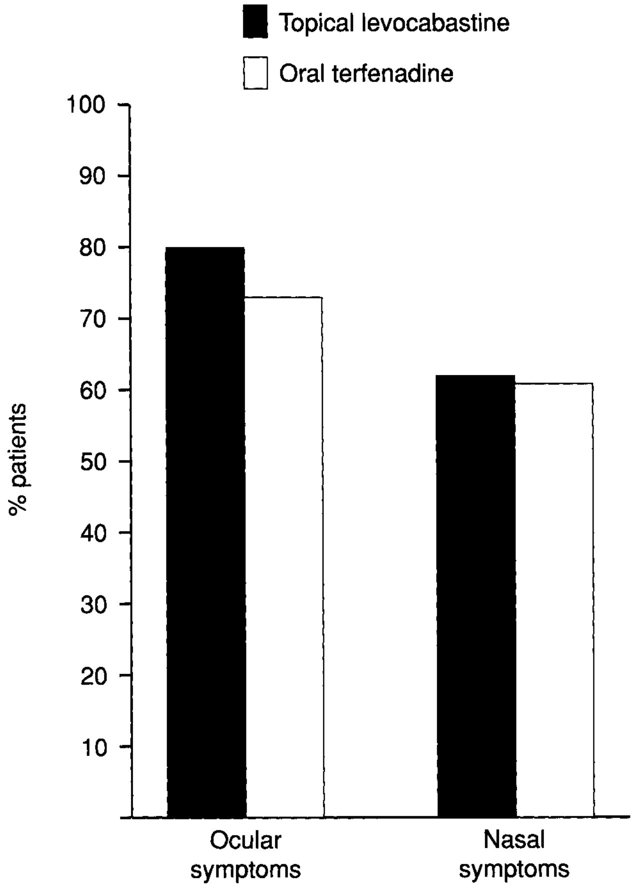

FIG. 1. Percentages of patients reporting excellent or good global therapeutic efficacy for ocular and nasal symptoms after 8 weeks of treatment with topical levocabastine or oral terfenadine.[33] Reproduced with the kind permission of Blackwell Scientific Publishers, London, UK.

good in 80% of levocabastine-treated patients compared with 73% in those who received terfenadine, with the effect of therapy on nasal symptoms being excellent or good in 62% and 61% of patients in the two groups, respectively (Fig. 1).[33]

Similarly, twice daily treatment with levocabastine eye drops and nasal spray has been shown to be at least as effective as oral loratadine (10 mg) once daily in a recent double-blind, primary care based trial involving 95 patients with seasonal allergic rhinoconjunctivitis.[36] A non-significant trend in favour of the topical antihistamine was apparent at the end of the 5-week treatment period, with an excellent or good response to therapy observed in 86% of levocabastine-treated patients compared with 77% for those who received loratadine.

As a double-dummy technique was used in these trials to blind study medication, patients treated with oral $H_1$-receptor antagonists also received placebo eye drops and nasal spray. Response rates as high as 73% have been associated with the use of topical placebos,[37] which may have contributed in part to the clinical ben-

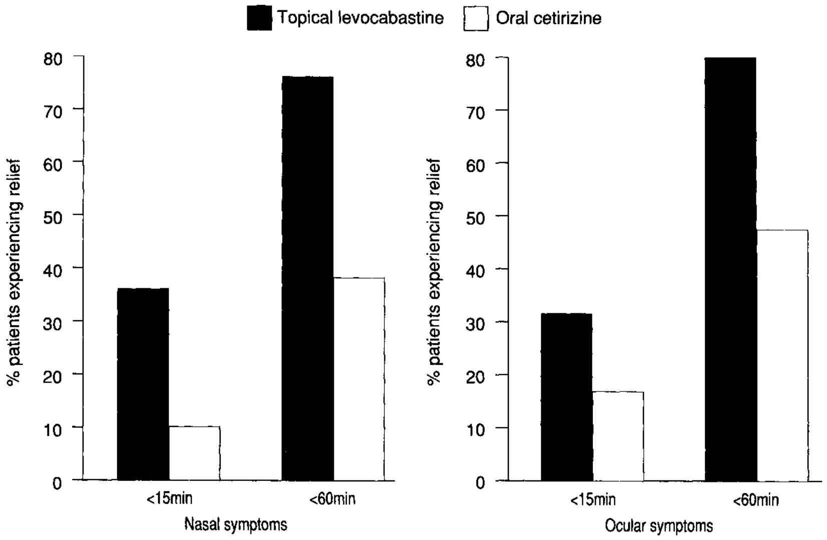

FIG. 2. Onset of action for nasal and ocular symptoms in patients receiving levocabastine nasal spray or oral cetirizine.[38]

efits seen in patients on oral antihistamines in these trials. Consequently, in order to obtain a more realistic assessment of the comparative efficacy of topical and oral antihistamine therapy, an open-label, randomized trial has recently been undertaken to compare the effects of twice daily treatments with levocabastine nasal spray, with eye drops on demand, with once daily oral cetirizine (10 mg) in more than 200 patients with perennial allergic rhinoconjunctivitis.[38] Treatment duration was 2 weeks.

As might be expected from the route of drug administration, onset of action was found to be significantly more rapid in patients treated with topical levocabastine than in those who received oral cetirizine ($p < 0.001$). In all, 36% of levocabastine-treated patients reported relief from nasal symptoms within 15 min of drug administration compared with 10% of those on cetirizine. Corresponding values for ocular symptoms were 32% and 17% in the two groups, respectively, at this time (Fig. 2). Therapeutic efficacy was generally found to be comparable in the two treatment groups, with no statistically significant intergroup differences reported during the course of the trial. In all, 61% of levocabastine-treated patients rated global therapeutic efficacy to be excellent or good compared with 62% on cetirizine.

*Levocabastine and azelastine:* The comparative efficacy of levocabastine nasal spray (0.5 mg/ml, two puffs per nostril twice daily) and topical azelastine (1 mg/ml, one puff per nostril twice daily) in the treatment of allergic rhinitis has been evaluated in a total of 242 patients in an open-label, randomized trial.[39] Treatment duration was 1 week. Onset of action was found to be comparable for the two topical antihistamines, with over 50% of patients in each group reporting significant relief from symptoms within 30 min of study drug administration. In general, therapeutic efficacy was also similar in the two treatment groups with a comparable reduction in the severity of all symptoms, including nasal congestion, seen in both treatment groups. Assessments of global therapeutic efficacy revealed a non-significant trend in favour of levocabastine, with the effect of treatment considered to be excellent or

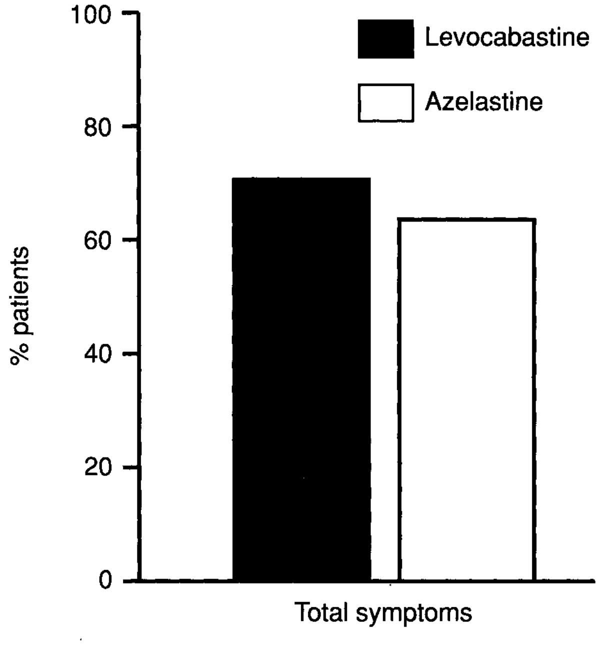

FIG. 3. Percentages of patients reporting excellent or good global therapeutic efficacy after 1 week of treatment with levocabastine or azelastine.[39]

good in 70% of patients who received levocabastine compared with 63% of those on azelastine (Fig. 3).

*Levocabastine and topical corticosteroids:* Twice daily treatment with levocabastine eye drops and nasal spray appears to be as effective as twice daily flunisolide nasal spray for the treatment of nasal symptoms of allergic rhinoconjunctivitis, according to the results of a randomized, parallel-group, single-blind study involving a total of 66 patients.[40] At the end of the 1 month treatment period, response to therapy was found to be excellent or good in 53% of levocabastine-treated patients compared with 64% of those who received the topical corticosteroid (N.S.). In this study, corticosteroid-treated patients also received naphazoline/antazoline eye drops four times daily. Levocabastine was found to provide significantly greater relief from concurrent ocular symptoms than this vasoconstrictor/antihistamine combination, with an excellent or good response to therapy reported in 47% and 19% of patients in the two groups, respectively, at the end of the trial ($p < 0.05$).

It is well documented that oral antihistamines are generally less effective than topical corticosteroids for the relief of nasal congestion.[41,42] This is not surprising as other mediators such as kinins, prostaglandin $D_2$ and leukotrienes $C_4$ and $D_4$ are also known to increase nasal airway resistance.[43,44] In order to assess whether combination therapy with an intranasal steroid could provide additional clinical benefit to that seen with a topical antihistamine alone, the efficacy of beclomethasone diproprionate nasal spray was evaluated as add-on therapy in a double-blind trial in 44 patients with allergic rhinitis who were already receiving intranasal levocabastine.[45] As might be expected, the severity of nasal congestion was found to be significantly lower in patients receiving both agents ($p < 0.001$), however, combination therapy was not associated with any additional improvement in the severity of other symptoms of allergic rhinitis compared with that seen with levocabastine alone (Fig. 4).

*Levocabastine and sodium cromoglycate:* Levocabastine eye drops and nasal spray appear to be

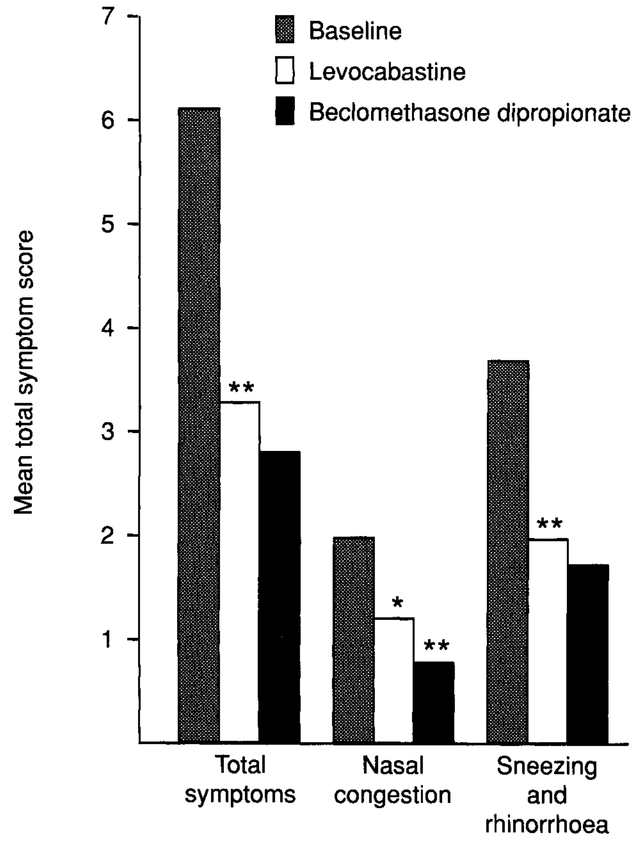

FIG. 4. Mean total symptom scores (0 = absent to 7 = severe) at baseline and after treatment with levocabastine nasal spray for 2 weeks followed by beclomethasone dipropionate nasal spray for 2 weeks. *$p < 0.05$; **$p < 0.001$ versus baseline.[45] Reproduced with the kind permission of Munksgaard Int. Publishers Ltd, Copenhagen, Denmark.

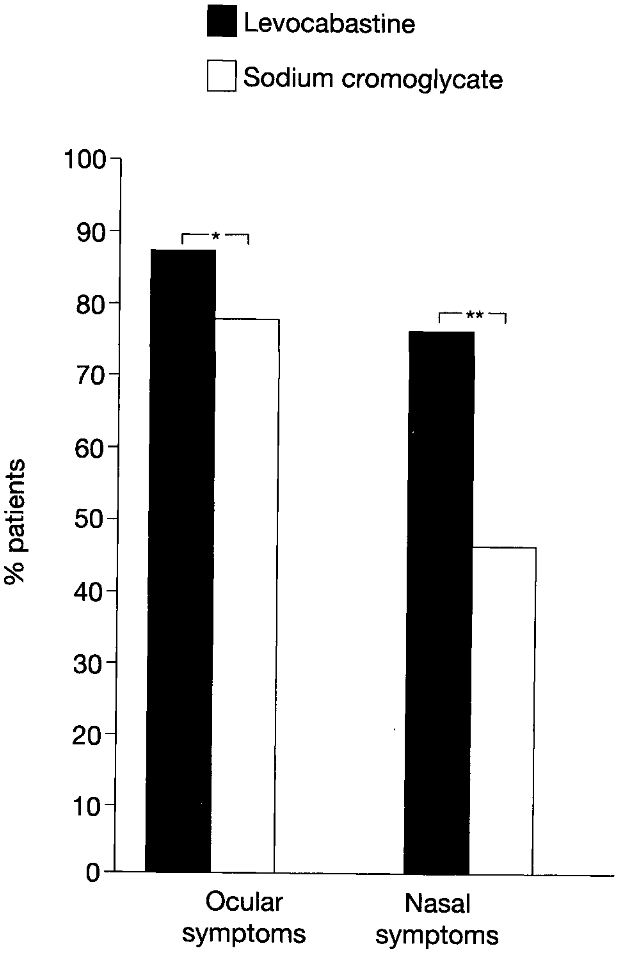

FIG. 5. Percentages of patients reporting excellent or good global therapeutic efficacy after 2 weeks of topical treatment with levocabastine or sodium cromoglycate.[46–49] Reproduced with permission.

significantly more effective for the treatment of allergic rhinoconjunctivitis than topical therapy with sodium cromoglycate (Fig. 5). To date, two double-blind trials, involving a total of 114 patients with allergic rhinitis, have been published which compare the efficacy of levocabastine nasal spray (0.5 mg/ml; two puffs per nostril four times daily) with that of intranasal sodium cromoglycate (20 mg/ml; two puffs per nostril four times daily).[46,47] In both trials, levocabastine was found to be significantly more effective than sodium cromoglycate at the end of the 2-week treatment period, with an excellent or good response to therapy seen in 76% and 46% of patients in the two groups, respectively ($p < 0.01$). Levocabastine eye drops (0.5 mg/ml; one drop in each eye twice daily) also appear to provide significantly greater relief from symptoms of allergic conjunctivitis than ocular sodium cromoglycate (20 mg/ml; one drop in each eye four times daily). In two double-blind, placebo-controlled studies involving a total of 158 patients, an excellent or good response to therapy was observed in 87% of levocabastine-treated patients compared with 78% in those who received sodium cromoglycate ($p < 0.05$).[48,49]

### The Efficacy of Levocabastine in Children

The therapeutic efficacy of topical levocabastine for the treatment of allergic rhinoconjunctivitis in paediatric patients has been assessed in studies involving more than 300 children and a comprehensive review of the role of topical antihistamine therapy in this patient population has been published.[50] Clinical experience, to date, suggests that topical levocabastine is at least as effective and well tolerated as the current first-line therapy for the treatment of allergic rhinoconjunctivitis in children, sodium cromoglycate, both as monotherapy or in combination with oral antihistamines, with a number of statistically significant differences in therapeutic efficacy in favour of the topical antihistamine reported in the largest of these trials.[51]

### Tolerability

Levocabastine eye drops and nasal spray appear to be well tolerated with an adverse effect profile comparable with that seen with sodium cromoglycate or placebo.[52] As might be expected from the route of drug administration, application site reactions are the most frequent adverse effect associated with topical levocabastine. In large scale clinical trials, local irritation following application has been reported in 3% of patients treated with levocabastine nasal spray and 14% of those who have received levocabastine eye drops. This compares with incidences of 4% and 15% for topical placebos, and 5% and 16% for sodium cromoglycate nasal spray and eye drops, respectively.

Levocabastine appears to be better tolerated than azelastine. Results of the only comparative study of levocabastine and azelastine undertaken to date reveal significantly higher incidences of application site reactions and taste disturbances in azelastine-treated patients than in those who received levocabastine (5% versus 1%; $p < 0.05$, and 5% versus 0%; $p < 0.01$, respectively).[39] Levocabastine also appears to be better tolerated than other topical therapeutic approaches, with local irritation following administration reported in up to 36% of patients receiving the vasoconstrictor/antihistamine eye drops naphazoline/antazoline, and 25% of those treated with the intranasal steroid flunisolide.[40]

Long-term ocular administration of levocabastine appears to be well tolerated, with comprehensive ophthalmological examinations in patients receiving levocabastine eye drops revealing no serious adverse effects to date.[53] Similarly, there has been no evidence of tachyphylaxis or rebound in patients receiving levocabastine eye drops, to date, with treatment durations of up to 4 months.[54]

In addition, it is important that a nasal spray should not affect ciliary function. The effects of levocabastine nasal spray on ciliary function have been studied *in vitro* on human adenoid tissue and *in vivo* in healthy volunteers. Levocabastine was not found to affect either ciliary beat frequency or mucociliary transit time suggesting that ciliotoxicity during long-term intranasal administration is extremely unlikely.[55]

### The Role of Levocabastine

An understanding of the immunological events during and after allergen exposure is necessary for the optimal clinical management of allergic rhinoconjunctivitis. As in bronchial asthma, a step-wise approach to therapy appears to be indicated depending on symptom frequency and severity.

The basic approach in the clinical management of any allergic disorder is environmental control. Following identification of the causative allergen, measures to reduce allergen exposure and appropriate patient education are essential. However, in many patients, complete allergen avoidance may be difficult to achieve. Specific immunotherapy may also be beneficial if the causative allergen is well defined, especially in moderate to severe pollen allergy, however the individual response to therapy with other allergens is highly variable. Consequently, almost all patients with allergic rhinoconjunctivitis will also require appropriate anti-allergic medication.

The wide range of pharmacological approaches available for the clinical management of allergic rhinoconjunctivitis enables treatment to be individualized according to patient needs. Oral $H_1$-receptor antagonists are generally considered as first-line therapy due to the central role of histamine $H_1$-mediated effects in the pathogenesis of this allergic condition.[12,13] However, a potent topical antihistamine with a rapid onset of action, such as levocabastine, may be a more appropriate therapeutic choice. Topical levocabastine appears to be at least as effective as oral antihistamines for the treatment of allergic rhinoconjunctivitis,[33] with the advantage of a significantly more rapid onset of action[38] and, in particular, may provide greater relief from symptoms on days with high pollen counts.[56] Furthermore, topical levocabastine is at least as effective as azelastine nasal spray, with the advantage of a more favourable tolerability profile and the availability of eye drops for the relief of concurrent ocular symptoms.[39]

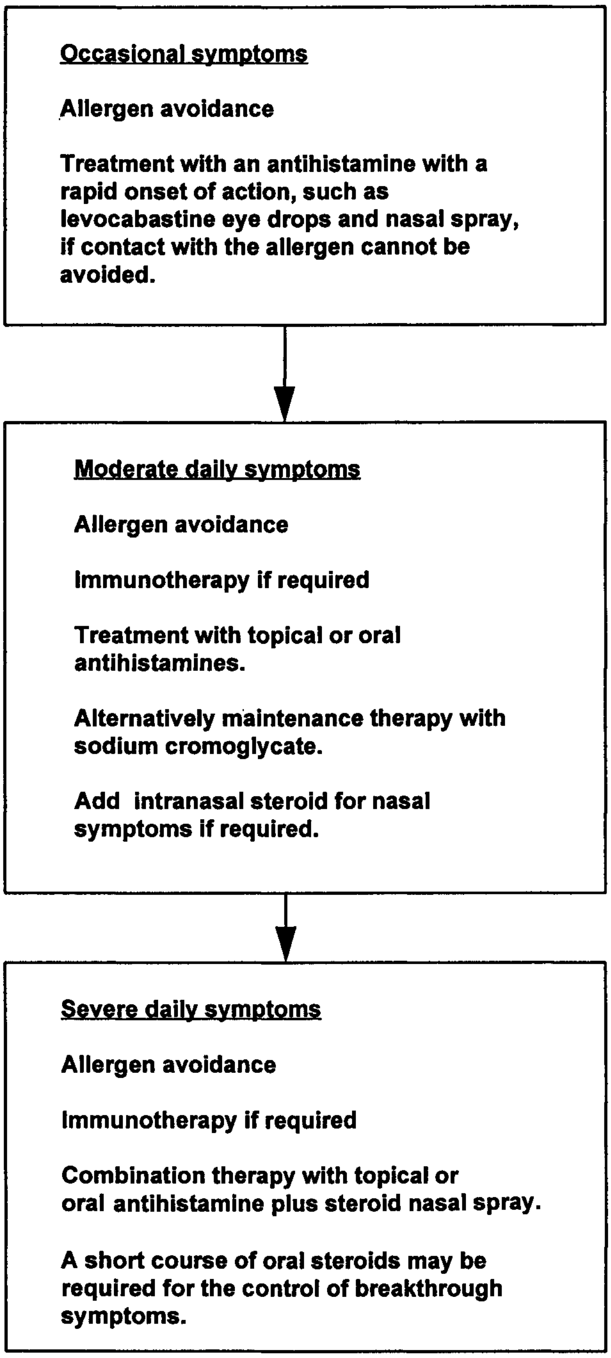

FIG. 6. Proposed algorithm for the treatment of allergic rhinoconjunctivitis.

It is well documented that nasal congestion is generally less responsive to treatment with $H_1$-receptor antagonists than other symptoms of allergic rhinoconjunctivitis.[41,42] Although preliminary data suggest that topical levocabastine may provide more effective relief from nasal symptoms than oral antihistamine therapy,[34,35] concurrent treatment with an intranasal steroid, or a topical or oral decongestant, may be indicated in patients with more severe daily symptoms, particularly if nasal congestion is predominant. The available data suggest that addition of an intranasal steroid provides significantly greater relief from nasal congestion than treatment with levocabastine nasal spray alone.[45]

Sodium cromoglycate is often considered as a primary treatment option for the treatment of allergic rhinoconjunctivitis in children due to its excellent adverse-effect profile. Results from paediatric trials suggest that levocabastine may be a more appropriate first-line therapy in this patient population being significantly more effective and as well tolerated.[50,51]

A proposed algorithm for the treatment of allergic rhinoconjunctivitis is shown in Fig. 6. It is apparent that the availability of an effective and well-tolerated topical antihistamine, such as levocabastine, is an important advance which broadens the range of therapeutic approaches available for the clinical management of allergic rhinoconjunctivitis. Clinical evidence available to date suggests that levocabastine is an attractive alternative to oral antihistamines and that this topical antihistamine should be considered as a first-line therapeutic option for the treatment of this atopic condition.

### References

1. Weeke ER. Epidemiology of hay fever and perennial allergic rhinitis. *Monogr Allergy* 1987; **21:** 1–20.
2. Åberg N. Asthma and allergic rhinitis in Swedish conscript. *Clin Exp Allergy* 1989; **19:** 59–63.
3. Bråbeck L, Kalvesten L. Urban living as a risk factor for atopic sensitization in Swedish schoolchildren. *Pediatr Allergy Immunol* 1991; **2:** 14–19.
4. Ishizaka T, Koimuza K, Ikemori R, *et al.* Studies of prevalence of Japanese cedar pollinosis among the residents in a densely cultivated area. *Ann Allergy* 1987; **58:** 265–270.
5. Von Mutius E, Martinez FD, Fritzsch C, *et al.* Prevalence of asthma and atopy in two areas of West and East Germany. *Am J Respir Crit Care Med* 1994; **149:** 358–364.
6. Juniper EF, Guyatt GH. Development and testing of a new measured health status for clinical trials in rhinoconjunctivitis. *Clin Exp Allergy* 1991; **21:** 77–83.
7. US Department of Health and Human Services. Asthma and allergies: an optimistic future. Bethesda: Dept of Health and Human Services. 1980: DHHS publication no. NIH80-388.
8. Cornell JT. Quantitative intranasal pollen changes. 3. The priming effect. *J Allergy* 1969; **43:** 33–44.
9. Togias A, Naclerio RM, Proud D, *et al.* Studies on the allergic and non-allergic nasal inflammation. *J Allergy Clin Immunol* 1988; **81:** 782–790.
10. Reed CE, Marcoux MD, Welsh PW. Effects of topical nasal treatment on asthma symptoms. *J Allergy Clin Immunol* 1988; **81:** 1042–1047.
11. Corren J, Aadinoff AD, Buchmeier AD. Nasal beclomethasone prevents the seasonal increase in bronchial responsiveness in patients with allergic rhinitis and asthma. *J Allergy Clin Immunol* 1992; **90:** 250–256.
12. Andersson M, Greiff L, Svensson C. Allergic rhinoconjunctivitis: the role of histamine. *Med Inflamm* 1994; **3:** 171–175.
13. Badhwar AK, Druce HM. Allergic rhinitis. *Clin Allergy* 1992; **76:** 789–803.
14. Simons FER. $H_1$-receptor antagonists: clinical pharmacology and therapeutics. *J Allergy Clin Immunol* 1989; **84:** 845–861.
15. Simons FER. $H_1$-receptor antagonists. Comparative tolerability and safety. *Drug Safety* 1994; **10:** 350–380.
16. Nizami RM. Treatment of ragweed allergic conjunctivitis with 2% cromolyn solution in unit doses. *Ann Allergy* 1981; **47:** 5–7.
17. Stafford CT. Allergic rhinitis: a useful guide to diagnosis and treatment. *Postgrad Med J* 1987; **81:** 147–157.
18. Friedlaender MH. Current concepts in ocular allergy. *Ann Allergy* 1991; **67:** 5–13.
19. Friedlaender MH. Corticosteroid therapy of ocular inflammation. *Int Ophthalmol* 1983; **23:** 175–182.
20. Vanden Bussche G. Levocabastine hydrochloride. *Drugs Future* 1986; **11:** 841–843.
21. Awouters F, Niernegers C, Janssen T, *et al.* Levocabastine: pharmacological profile of a highly effective inhibitor of allergic reactions. *Agents Actions* 1991; **35:** 12.
22. Van Wauwe JP. Animal pharmacology of levocabastine: a new type of $H_1$-antihistamine well-suited for topical application. In: Mygind N, Naclerio RM, eds. *Rhinoconjunctivitis: New Perspectives in Topical Treatment of Seasonal Allergic Rhinitis.* Proceedings of the XIIIth International Congress of Allergology and Clinical Immunology. Gottingen: Hogrefe and Huber, 1989; 27–34.
23. Megens AAHP, Vermeire J, Awouters FHL. Protection from compound 48/80-induced shock in rats: comparison of nine recent antihistamines with levocabastine. *Life Sci Advances Pharmacol* 1995; in press.
24. Heykants JJP, Snoeck E, Awouters F, Van Peer A. Antihistamines. In: Van Boxel CJ, Holford NHG, Danhof N, eds. The *in vivo* study of drug action. Amsterdam: Elsevier Science, 1992; 337–356.
25. Dechant KL, Goa KL. Levocabastine. A review of its pharmacological properties and therapeutic potential as a topical antihistamine in allergic rhinitis and conjunctivitis. *Drugs* 1991; **41:** 202–224.
26. Pécoud A, Zuber P, Kelly M. Effect of a new selective $H_1$-receptor antagonist (levocabastine) in a nasal and conjunctival provocation test. *Int Arch Allergy Appl Immunol* 1987; **82:** 541–543.
27. Abelson MB, Smith LM. Levocabastine: evaluation in the histamine and compound 48/80 models of ocular allergy in humans. *Ophthalmology* 1988; **95:** 1494–1497.
28. Palma-Carlos AG, Palma-Carlos ML, Rombaut N. The effect of levocabastine nasal spray in nasal provocation tests. *Int J Clin Pharmacol Res* 1988; **8:** 25–30.
29. Rimås M, Kjellman N-IM, Blychert L-O, Björkstén B. Topical levocabastine protects better than sodium cromoglycate and placebo in conjunctival provocation tests. *Allergy* 1990; **45:** 18–21.
30. Stokes TC, Feinberg G. Rapid onset of action of levocabastine eye drops in histamine-induced conjunctivitis. *Clin Exp Allergy* 1993; **23:** 791–794.
31. de Graaf-in't Veld C, Garrelds IM, van Toorenenbergen AW, *et al.* Effect of topical levocabastine on nasal response to allergen challenge and nasal hyperreactivity in patients with perennial rhinitis. *Ann Allergy* 1995; in press.
32. Tomiyama S, Ohnishi M, Okuda M. The dose and duration of effect of levocabastine, a new topical $H_1$-receptor antagonist, on nasal provocation reaction to allergen. *Am J Rhinology* 1993; **7:** 85–88.
33. Bahmer FA. Topical levocabastine—an effective alternative to oral antihistamines in seasonal allergic rhinoconjunctivitis. *Clin Exp Allergy* 1995; **25 (3):** 220–227.
34. Bahmer FA, Ruprecht KW. Safety and efficacy of topical levocabastine compared with oral terfenadine. *Ann Allergy* 1994; **72:** 429–434.
35. Søhoel P, Freng BA, Kramer J, *et al.* Topical levocabastine compared with oral terfenadine for the treatment of seasonal allergic rhinoconjunctivitis. *J Allergy Clin Immunol* 1993; **23:** 791–794.
36. The Swedish GP Allergy Team. Topical levocabastine compared with oral loratadine for the treatment of seasonal allergic rhinoconjunctivitis. *Allergy* 1994; **49:** 611–615.
37. Leino M, Ennevaara K, Latvala A-L, *et al.* Double-blind group comparative study of 2% nedocromil sodium eyedrops with 2% sodium cromoglyccate and placebo eyedrops in the treatment of seasonal allergic conjunctivitis. *Clin Exp Allergy* 1992; **22:** 929–932.
38. Drouin MA, Yang WH, Horak F. Faster onset of action with topical levocabstine than with oral cetirizine. *Med Inflamm* 1995; **4 (1):** S5–S10.
39. Mösges R, Spaeth J, Klimek L. Efficacy and tolerability of levocabastine and azelastine nasal sprays for the treatment of allergic rhinitis. *Med Inflamm* 1995; **4 (1):** S11–S15.
40. Bende M, Pipkorn U. Topical levocabastine, a selective $H_1$-receptor antagonist in seasonal allergic rhinoconjunctivitis. *Allergy* 1987; **42:** 512–525.
41. Busse W. New directions and dimensions in the treatment of allergic rhinitis. *J Allergy Clin Immunol* 1988; **82:** 890–900.
42. Delafuente JC, Davis TA, Davis JA. Pharmacotherapy of allergic rhinitis. *Clin Pharmacy* 1989; **8:** 474–485.
43. Doyle WJ, Boehm S, Skoner DP. Physiologic responses to intranasal dose-response challenges with histamine, methacholine, bradykinin and prostaglandin in adult volunteers with and without nasal allergy. *J Allergy Clin Immunol* 1990; **86:** 924–935.
44. Rajakalasingam K, Polosa R, Lau LCK, *et al.* Comparative nasal effects of bradykinin and histamine: influence on nasal airways resistance and plasma protein exudation. *Thorax* 1993; **48:** 324–329.
45. Van de Heyning PH, Van Haesendock J, Creten W, Rombaut N. Effect of topical levocabastine on allergic and non-allergic perennial rhinitis. *Allergy* 1988; **43:** 386–391.
46. Palma-Carlos AG, Chiera C, Conde TA, Robalo Corderio JA. Double-blind comparison of levocabastine nasal spray with sodium cromoglycate nasal spray in the treatment of seasonal allergic rhinitis. *Ann Allergy* 1991; **67:** 394–398.
47. Schata M, Jorde W, Richarz-Barthauer U. Levocabastine nasal spray better than sodium cromoglycate and placebo in the topical treatment of seasonal allergic rhinitis. *J Allergy Clin Immunol* 1991; **87:** 873–878.
48. Azevedo M, Castel-Branco MG, Ferrez Oliveira J, *et al.* Double-blind comparison of levocabastine eye drops with sodium cromoglycate and placebo in the treatment of seasonal allergic conjunctivitis. *Clin Exp Allergy* 1991; **21:** 689–694.
49. Davies BH, Mullins, J. Topical levocabastine is more effective than sodium cromoglycate for the prophylaxis and treatment of seasonal allergic conjunctivitis. *Allergy* 1993; **48:** 519–524.
50. Möller C. Topical levocabastine. A new approach for the treatment of allergic rhinoconjunctivitis in children. *Allergy* 1995; in press.
51. Vermeulen J, Mercer M. Topical levocabastine is more effective than sodium cromoglycate in children with seasonal allergic rhinoconjunctivitis. *Pediatr Allergy Immunol* 1994; **5:** 209–213.
52. Howarth P. A Review of the tolerability and safety of levocabastine eye drops and nasal spray. Implications for patient management. *Med Inflamm* 1995; **4 (1):** S26–S30.
53. Janssens M, Blockhuys S. Tolerability of levocabastine eye drops. *Doc Ophthalmol* 1993; **84:** 111–118.
54. Frostad AB, Olsen AK. A comparison of topical levocabastine and sodium cromoglycate in treatment of pollen-provoked allergic conjunctivitis. *Clin Exp Allergy* 1993; **23:** 406–409.
55. Merkus FWHM, Schüster-van Hees MTIW. Influence of levocabastine suspension on ciliary beat frequency and mucociliary clearance. *Allergy* 1992; **47:** 230–233.
56. de Azevedo M. Topical levocabastine—a review of therapeutic efficacy on days with high pollen counts. *Med Inflamm* 1995; **4 (1):** S21–S25.
